# The correlation between graphene characteristic parameters and resonant frequencies by Monte Carlo based stochastic finite element model

**DOI:** 10.1038/s41598-021-02429-2

**Published:** 2021-11-25

**Authors:** Liu Chu, Jiajia Shi, Eduardo Souza de Cursi

**Affiliations:** 1 School of Transportation and Civil Engineering, Nantong University, Nantong, 226019 China; 2 Département Mécanique, Institut National des Sciences Appliquées de Rouen, 76801 Rouen, France

**Keywords:** Mechanical and structural properties and devices, Computational science, Computational methods

## Abstract

The uncertainty and fluctuations in graphene characteristic parameters are inevitable issues in both of experimental measurements and numerical investigations. In this paper, the correlations between characteristic parameters (Young’s modulus, Poisson’s ratio and thickness of graphene) and resonant frequencies are analyzed by the Monte Carlo based stochastic finite element model. Based on the Monte Carlo stochastic sampling procedure, the uncertainty in the characteristic parameters are properly propagated and quantified. The displacements and rotation modes of graphene under the resonant vibration computed by the finite element method are verified. Furthermore, the result robustness of stochastic samples is discussed based on the statistic records and probability density distributions. In addition, both the Pearson and Spearman correlation coefficients of the corresponding characteristic parameters are calculated and compared. The work in this paper provides a feasible and highly efficient method for the characteristic parameter correlation discussion by taking uncertainty into consideration.

## Introduction

Graphene is a 2D array of carbon atoms with sp2 covalent bonds to form honeycomb cells. The numerical models and analytical methods are effective alternatives to the real experiments with the merits in time and cost saving^1^. The most popular and widely used methods for mechanical analysis of graphene are the molecular mechanics (MM), molecular dynamics (MD) and finite element model (FEM)^2^. In the MM algorithms, the equilibrium configurations of the molecular domain are obtained by minimising energy, while in the MD algorithms, the momentum equations involving interatomic forces are integrated over time. Compared with the other two methods, FEM is more competitive in terms of computational efficiency, especially for large random sample spaces.

The Monte Carlo based stochastic finite element model (MC-SFEM) combines the Monte Carlo stochastic sampling method with the FEM to expand applications in uncertainty propagation^3^. For example, the MC-SFEM is an effective method to discuss and analyze the mechanical impacts of random distributed defects in graphene by mapping the stochastic series into the periodic lattice^4^. In addition, the uncertainty propagation of graphene material and geometrical parameters can be implemented by the MC-SFEM^5^. Also, the costs of repeated FEM computations for stochastic samples are much lower than that of MM and MD^2^. Moreover, more sophisticated attempts on the material spatial randomness are taken into consideration by the implementation of statistical to representative volume element^6^. The scale of homogenization is the mesoscale that separates the micro-heterogeneity from the macroscale. In addition, through the iterative and generalized stochastic perturbation approach, the differences between the numerical and semi-analytical homogenization methods in the context of geometrical uncertainty are presented^7^. Therefore, the MC-SFEM is an applicable and promising method for the low dimensional nanomaterial study, which deserves more exploration.

Even numerous related works have presented the extraordinary physical properties of graphene, the uncertainty and fluctuations in the characteristic parameters are inevitable issues in both of experimental measurements and numerical investigations. In terms of experimental measurements, a broad range of stiffness values from 0.27 TPa to 1.47 TPa were obtained in the tensile test^8^, with breaking strengths ranging from 3.6 GPa to 63 GPa. In addition, the Young’s modulus is extracted as 0.5 TPa^9^ in the measurement of the bending stiffness by the atomic force microscope (AFM) based on nanoindentation method. In another study, the Young’s modulus equals to 1.0 ± 0.1 TPa and the corresponding intrinsic stress is 130 ± 10 GPa at a strain of 0.25^10^. Therefore, it is necessary to take the uncertainty and fluctuations in graphene characteristic parameters into consideration and perform quantification research.

For the uncertainty quantification and propagation, the traditional FEM of graphene has limitations due to the deterministic values of characteristic parameters. Based on MC-SFEM, it is possible to extend the deterministic values into the interval ranges for the characteristic parameters of graphene. On the other hand, the complicated relationships between characteristic parameters and specific mechanical responses of graphene cannot be described by explicit expressions^11–32^. The correlation coefficients are the effective and feasible factors to analyze the sensitive impacts of characteristic parameters on mechanical responses. In terms of performing correlation coefficient computation, the MC-SFEM is an effective method to provide the sufficient stochastic samples in the broad interval ranges.

In this paper, the MC-SFEM is performed by the integration of stochastic sampling simulation with FEM computation for uncertainty quantification and propagation. The corresponding resonant frequencies of graphene are tracked and recorded. The basic deterministic FEM is verified by the comparison with the previous reported work. The robustness of results for the different stochastic sample space sizes is analyzed and discussed. The correlation coefficients of the Young’s modulus, Poisson’s ratio, thickness of graphene are calculated, respectively. A short conclusion is summarized in the last section.

## Model formation

### Characteristic parameters

**Figure 1:**
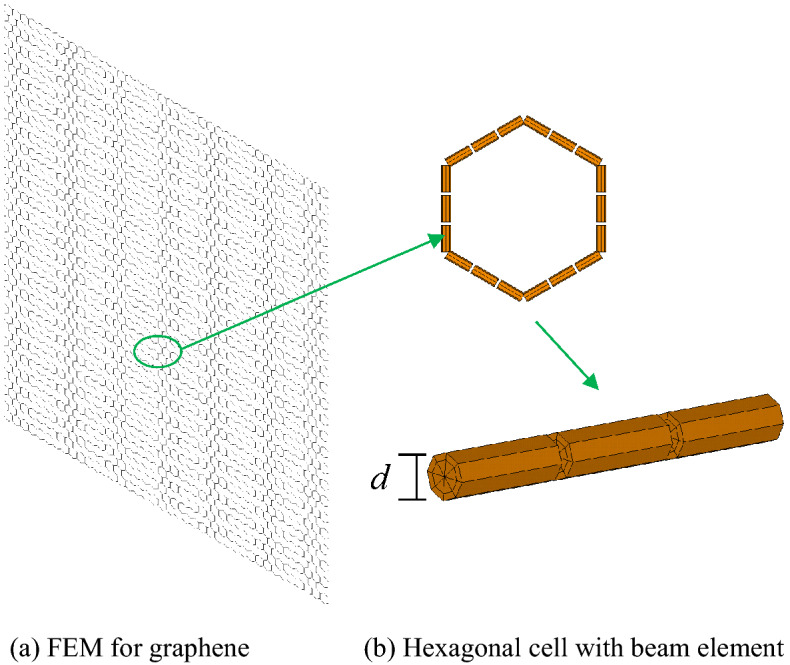
Graphene specific hexagonal cell in FEM.

According to the periodic honeycomb structure of graphene, the specific hexagonal cell in FEM is defined by geometrical and material parameters, including the length of carbon–carbon bonds, the thickness of the graphene (*d*), the Young’s modulus (*E*), the Possion’s ratio (*v*) and the physical density. The geometrical structure of graphene in FEM is presented in Fig. 1. The length of carbon–carbon covalent bonds represents the distance between two carbon atoms, which is also the side length of periodic hexagon cell. The interaction between two carbon atoms is represented by the beam connection. In addition, the thickness of the graphene is introduced as the diameter of the beam cross section as shown in Fig. 1b. Among the geometrical and material parameters of graphene, *d*, *E*, *v* are chosen as the characteristic parameters for the impact discussion and correlation coefficient computation, while the length of carbon–carbon bonds and the physical density are settled as the deterministic values as static references in the relative comparison.

**Figure 2:**
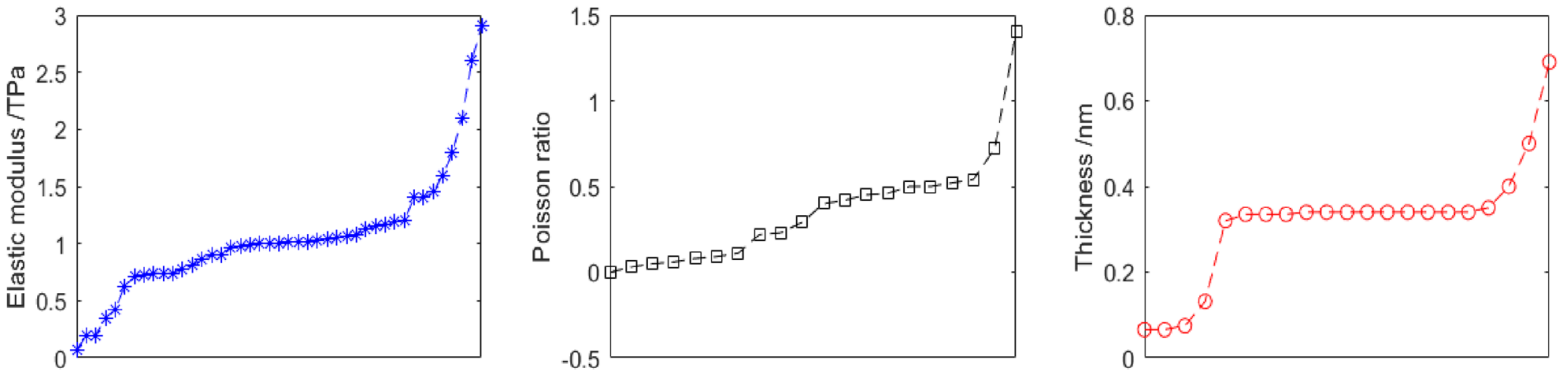
The specific values for the characteristic parameters of graphene in the related references.

In order to set precise interval ranges for the characteristic parameters, the related references are recorded in Table 1 and Fig. 2. Based on the statistic data in the references, the interval ranges are 0.05 to 3 TPa, 0.01 to 0.48, and 0.05 to 0.7 nm, for the Young’s modulus, Poission’s ratio, and the thickness of graphene, respectively.

**Table 1:** The related references for the characteristic parameters of graphene.

Source	E (TPa)	Type of FEM	Source	Thickness (nm)
*SLGS*^11^	1.03	SF	*Lee*^10^	0.335
*SLGS*^12^	1.17	SF	*Yakobson*^13^	0.066
*SLGS*^14^	0.99	SF	Hernandez^15^	0.34
*SLGS*^16^	1.08	SF	*Pantano*^17^	0.34
*SLGS*^18^	1.04	SF	*Pantano*^19^	0.075
*SLGS*^20^	1.00	SF	*Lu*^21^	0.34
*Flakes*^22^	1.45	SF	*Odegard*^23^	0.69
*GO*^24^	0.20	Coupled AFM/FEM	*Gupta*^25^	0.34
*SLGS*^26^	1.13	SF	*Wei*^27^	0.335
*SLGS*^28^	1.07	SF	*Song*^29^	0.34
*Nanoribbon*^30^	0.74	SpFEM	*Huang*^31^	0.335
*SLGS*^32^	0.74	SpFEM	*Zhang*^33^	0.066
*Nanoribbon*^34^	1.05	SpFEM	*Baykasoglu*^35^	0.34
*Nanoribbon*^36^	1.80	SF	*Chen*^37^	0.4
*SLGS*^38^	1.01	SF	*Zhou*^39^	0.34
*SLGS*^35^	0.35	Coupled MM/FEM	*Gil*^40^	0.32
*SLGS*^41^	1.19	SpFEM	*Arash*^42^	0.34
*SLGS*^43^	1.00	SF	*Niaki*^44^	0.34
*Pillared graphene*^45^	0.19	SF	*Hartmann*^46^	0.132
*SLGS*^47^	1.02	SF	*Jiang*^48^	0.35
*SLGS*^49^	1.60	SF	*Zhu*^50^	0.5
*SLGS*^51^	1.15	SF	**Source**	**Poisson’s ratio**
*DLGS*^52^	1.00	SF	*SLGS*^12^	0.5
*SLGS*^53^	0.98	SF	*SLGS*^18^	1.4
*MLGS*^54^	0.63	Alternative FEM	*SLGS*^20^	0.29
*SLGS*^55^	0.78	SF	*Flakes*^22^	0.72
*SLGS*^56^	1.20	SF	*SLGS*^32^	0.22
*TLGS*^57^	1.02	SpFEM	*Nanoribbon*^34^	0.54
*SLGS*^58^	2.90	Combined spring/beam elements	*Nanoribbon*^36^	0.46
*GO*^59^	0.07	SF	*SLGS*^38^	0.11
*GO*^60^	2.60	Shell element	*Pillared graphene*^46^	0.03
*SLGS*^61^	0.90	SF	*DLGS*^53^	0.09
*Graphyne*^62^	0.71	AFM-FEA	*MLGS*^55^	0.45
*SLGS*^63^	0.86	SF	*SLGS*^56^	0.5
*SLGS*^64^	2.10	SpFEM	*SLGS*^57^	0.05
*SLGS*^65^	0.81	SF	*GO*^61^	0.06
*SLGS*^66^	0.97	Tersoff-Bremmer FEM	*Graphyne*^63^	0.40
*SLGS*^67^	0.90	Axisymmetric shell model	*SLGS*^64^	0.52
*SLGS*^68^	1.40	Coupled MD-FEM	*SLGS*^65^	− 0.001
*SLGS*^69^	0.42	SpFEM	*Graphane*^70^	0.42
*SLGS*^71^	1.40	Shell element	*SLGS*^72^	0.08
*PGS*^73^	0.73

### Correlation coefficient computation

The estimated correlation matrix ***A*** is a symmetric matrix of the order $N_{{\text{var}}}$ and can be written as

1 $$A = I + L + L^{T} ,$$

where $I$ is the identity matrix and $L$ is the strictly lower triangular matrix with entries within the range $\left\langle { - 1,1} \right\rangle$. There are $N_{c}$ pairwise correlations:

2 $$N_{c} = \left( \begin{gathered} N_{{\text{var}}} \hfill \\ 2 \hfill \\ \end{gathered} \right) = \frac{{N_{{\text{var}}} (N_{{\text{var}}} - 1)}}{2}.$$

#### Pearson correlation coefficient

The most well-known correlation measurement is the linear Pearson correlation coefficient (PCC)^74^. The PCC takes values between − 1 and + 1, and provides an effective factor to evaluate the strength of the linear relationship between two variables. The actual PCC between two variables, $X_{i}$ and $X_{j}$, can be estimated using the sample correlation coefficient $A_{ij}$:

3 $$A_{ij} = \frac{{\sum\nolimits_{s = 1}^{{N_{sim} }} {({\text{x}}_{i,s} - \overline{{\text{X}}}_{i} )({\text{x}}_{j,s} - \overline{{\text{X}}}_{j} )} }}{{\sqrt {\sum\nolimits_{s = 1}^{{N_{sim} }} {({\text{x}}_{i,s} - \overline{{\text{X}}}_{i} )^{2} } \sum\nolimits_{s = 1}^{{N_{sim} }} {({\text{x}}_{j,s} - \overline{{\text{X}}}_{j} )^{2} } } }},$$

4 $$\overline{{\text{X}}}_{i} = \frac{1}{{N_{sim} }}\sum\limits_{s = 1}^{{N_{sim} }} {{\text{x}}_{i,s} } ,\quad \overline{{\text{X}}}_{j} = \frac{1}{{N_{sim} }}\sum\limits_{s = 1}^{{N_{sim} }} {{\text{x}}_{j,s} } ,$$

where ${\text{x}}_{i,s}$ is the actual data matrix, $s = 1,2, \ldots ,N_{sim}$ is the data sequence number of each vector, and $N_{sim}$ is the data sequence length of each vector. $i = 1,2, \ldots ,N_{{\text{var}}}$ is the variable index. In this study, $N_{{\text{var}}}$ equals to 3, with Young’s modulus ($i = 1$), Poisson’s ratio ($i = 2$) and thickness of graphene ($i = 3$).$s$ is directly related to the number of stochastic samples. $N_{sim}$ is settled as 10,000 in this work to have sufficiently huge sample spaces. $j = 1,2, \ldots ,N_{ord}$ is corresponding with the mode of resonant vibration. In addition. $\overline{{\text{X}}}_{i}$ is the mean of Young’s modulus, Poisson’s ratio and thickness of graphene; while $\overline{{\text{X}}}_{j}$ is computed by the mean of the jth order resonant frequencies of graphene.

When the actual data ${\text{x}}_{i,s}$ of each vector $i = 1,2, \ldots ,N_{{\text{var}}}$ are standardized into $z_{i,s}$ that yield zero averages and unit sample variance estimates, the formula can be simplified to

5 $$A_{ij} = \frac{1}{{N_{sim} }}\sum\limits_{s = 1}^{{N_{sim} }} {z_{i,s} z_{j,s} } ,$$

where $z_{i,s} = \frac{{X_{i} - \overline{X}_{i} }}{{\sigma_{{X_{i} }} }},$ $z_{j,s} = \frac{{X_{j} - \overline{X}_{j} }}{{\sigma_{{X_{j} }} }}$. The mean of variables can also be written as $\overline{X}_{i} = \mu_{{X_{i} }} = E(X_{i} )$ and the variances are computed as $\sigma_{{X_{i}^{2} }} = E[(X_{i} - E(X_{i} ))^{2} ] = E(X_{i}^{2} ) - E^{2} (X_{i} )$. Therefore, the sample correlation coefficient can be expressed as

6 $$A_{ij} = \frac{{E(X_{i} X_{j} ) - E(X_{i} )E(X_{j} )}}{{\sigma_{{X_{i} }} \sigma_{{X_{j} }} }}.$$

#### Spearman correlation coefficient

The formula for Spearman correlation coefficient^75^ estimation is identical to the one for Pearson linear correlation except that the values of random variables $X_{i}$ and $X_{j}$ are replaced by the ranks $\pi_{i,s}$ and $\pi_{j,s}$, $s = 1,2, \ldots ,N_{sim}$. The ranks are permutation of numbers. It is convenient to transform the ranks into

7 $$r_{i,s} = \pi_{i,s} - \overline{\pi }_{i} ,\quad r_{j,s} = \pi_{j,s} - \overline{\pi }_{j} .$$

The rank correlation is then defined as,

8 $$A_{ij} = \frac{{\sum {r_{i,s} r_{j,s} } }}{{\sqrt {\sum {r_{i,s}^{2} \sum {r_{j,s}^{2} } } } }}.$$

By noting that the sum of the first $N_{sim}$ squared integers is $\frac{{N_{sim} (N_{sim} + 1)(2N_{sim} + 1)}}{6}$, we find that $\sum {r_{i,s}^{2} = \sum {r_{j,s}^{2} } } = \frac{{N_{sim}^{3} - N_{sim} }}{12}$, and the rank correlation is,

9 $$A_{ij} = \frac{{12\sum {r_{i,s} r_{j,s} } }}{{N_{sim} (N_{sim}^{2} - 1)}} = \frac{{12\sum {\pi_{i,s} \pi_{j,s} } }}{{N_{sim}^{3} - N_{sim} }}\;\; - 3\frac{{N_{sim} + 1}}{{N_{sim} - 1}}.$$

Another formula exists for Spearman correlation suitable for data with no ties. The correlation coefficient between any two vectors that each consisting of permutations of integer ranks from 1 to $N_{sim}$ is,

10 $$A_{ij} = 1 - \frac{6D}{{N_{sim} (N_{sim}^{2} - 1)}},$$

where $D$ is the sum of square of $d_{s} = s - \pi_{s}$, which is the differences between the $s$th integer elements in the vectors,

11 $$D = \sum\limits_{s = 1}^{{N_{sim} }} {d_{s}^{2} } .$$

Every mutual permutation of ranks can be achieved by permuting the ranks $\pi_{s}$ of the second variable against the identity permutation corresponding to that of the first variable. Therefore,

12 $$D = \sum\limits_{s = 1}^{{N_{sim} }} {(s - \pi_{s} )^{2} } = 2\left[ {\sum\limits_{s = 1}^{{N_{sim} }} {s^{2} } - \sum\limits_{s = 1}^{{N_{sim} }} {(s\pi_{s} )} } \right].$$

Meanwhile, it can be obtained that

13 $$D = \frac{{N_{sim} (N_{sim} + 1)(2N_{sim} + 1)}}{3} - 2\sum\limits_{s = 1}^{{N_{sim} }} {(s\pi_{s} )} .$$

The lowest correlation is achieved for the reverse ordering of rank numbers and corresponds to the case when the sum D equals to $\frac{{N_{sim} (N_{sim}^{2} - 1)}}{3}$. In turn, the maximum correlation is achieved for identical ranks and the sum equals zero.

### Monte Carlo based stochastic finite element model

**Figure 3:**
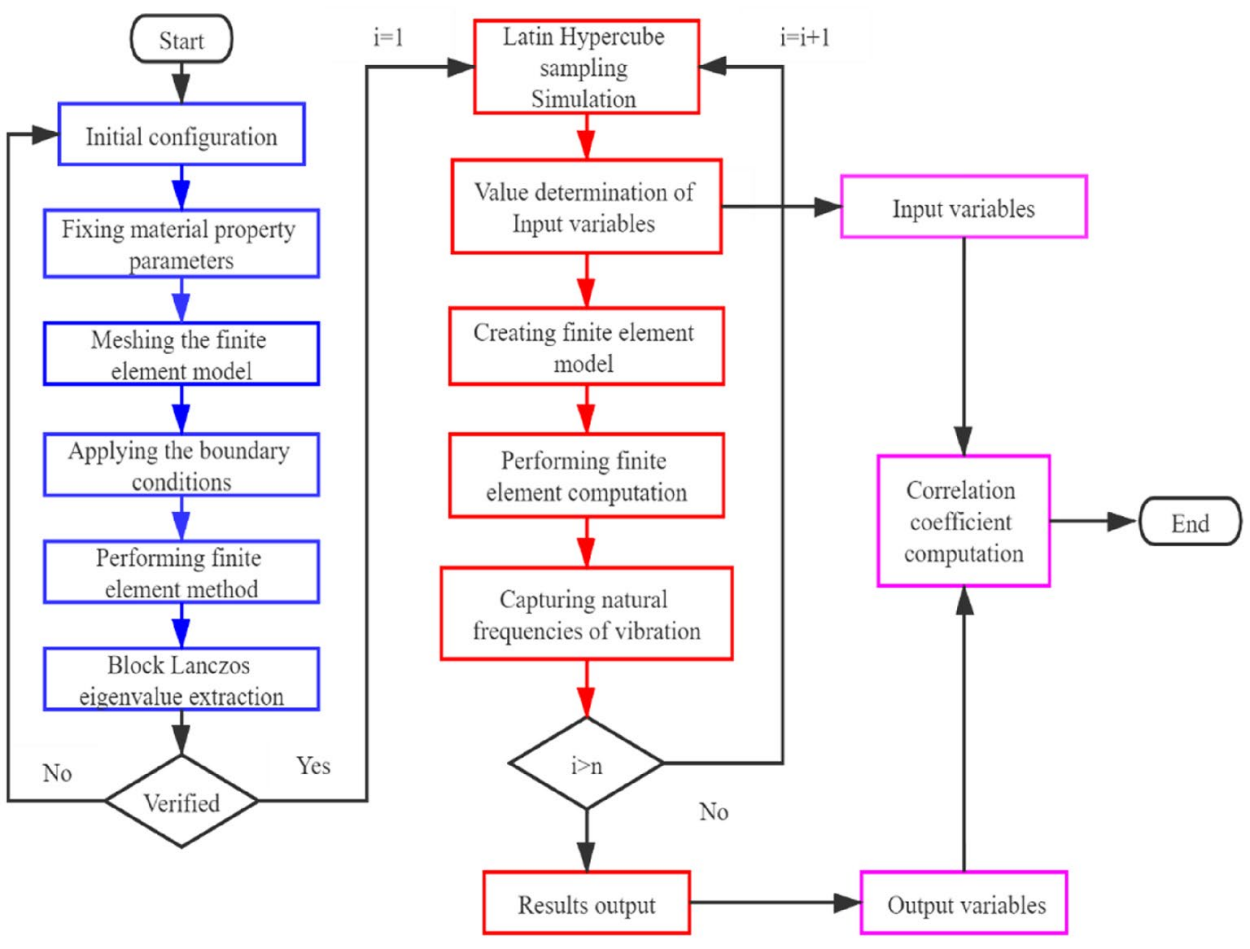
The flowchart of the MC-SFEM for correlation coefficient computation.

For the program implementation of MC-SFEM, two modules are compiled as presented in Fig. 3 with two different colors to remark. The process of finite element computation for the resonant frequencies of graphene is presented in the blue blocks in the flowchart, while the stochastic sampling procedure is presented in the red blocks. Finally, the input and output data are transferred into the correlation coefficient computation. To be clear, the integration of the Monte Carlo stochastic sampling procedure with the finite element computation is concluded in seven steps:

Step 1: Define the initial configuration of graphene with the periodic hexagonal lattice, such as the thickness of graphene (diameter of the section in beam elements), which is stochastically sampled as a variable in the following process. The distance between the two carbon atoms and the number of hexagons in the longitude and latitude sides of graphene are defined with the deterministic values.

Step 2: Introduce material characteristic parameters, among which the Young’s modulus and Poisson’s ratio are also the variables in the Monte Carlo stochastic sampling procedure.

Step 3: Mesh the beam elements and define the boundary conditions, where the four edges of graphene are fixed, the six freedom degrees (displacements and rotations in x, y, z directions) of each node in the four edges of graphene are settled to be zeros.

Step 4: Perform the finite element computation and use the Block Lanczos eigenvalue extraction for the resonant frequency calculation. The validation of the deterministic FEM is the premise of the following MC-SFEM.

Step 5: Apply the Monte Carlo stochastic sampling simulation to propagate the parameter uncertainty within specific intervals by the uniform probability distribution. Besides, the values of the characteristic parameters are recorded and stored as the input data for correlation analysis.

Step 6: Repeat the FEM computation and transfer the resonant frequencies of graphene as the output data.

Step 7: Based on the huge stochastic sample spaces of input and output data, the Pearson correlation coefficient and Spearman correlation coefficient are calculated.

It is important to be mentioned that for the Monte Carlo stochastic sampling method in Step 5, the Latin Hypercube sampling (LHS) method is performed to ensure the sampling efficiency and reduce computational costs. By dividing the range of each variable into disjoint intervals with equal probabilities, the samples are randomly selected from each interval. The LHS method effectively avoids the point clustering and duplication^76^, especially for the uniform probability density distribution in this study. Therefore, LHS procedure is used for random sample generation in the corresponding intervals of the characteristic parameters.

In addition, other probabilistic methods applied in the homogenization method are available alternatives as shown in literatures^77–80^. However, the uniform probability distribution is selected and performed in this study for several reasons. On the one hand, uncertainty and fluctuations in the characteristic parameters present broad interval ranges without deterministic probability density distributions. The uniform probability distribution is more comprehensive to fairly take each possible value in the certain intervals into consideration. On the other hand, the uniform probability distribution is more effective to provide stochastic samples for the correlation coefficient computation, since each possible value is unbiased sampled. Therefore, compared to the commonly used Gaussian, Weibull, Poisson, etc. probabilistic distributions, the uniform probability density distribution is more feasible to propagate the uncertainty for the characteristic parameters of graphene.

## Model validation

Based on the program flowchart, the deterministic FEM of graphene is created by the ANSYS Parameter Design Language (Version 14.5, APDL, ANSYS, USA). The BEAM188 element is chosen to mesh the carbon–carbon bonds in graphene. Beam 188 element is suitable for analyzing slender to moderately stubby/thick beam structures. Each node of Beam 188 element has six degrees of freedom. In addition, Beam 188 element is based on the Timoshenko beam theory which is effective in resonant frequency computatioin.

**Figure 4:**
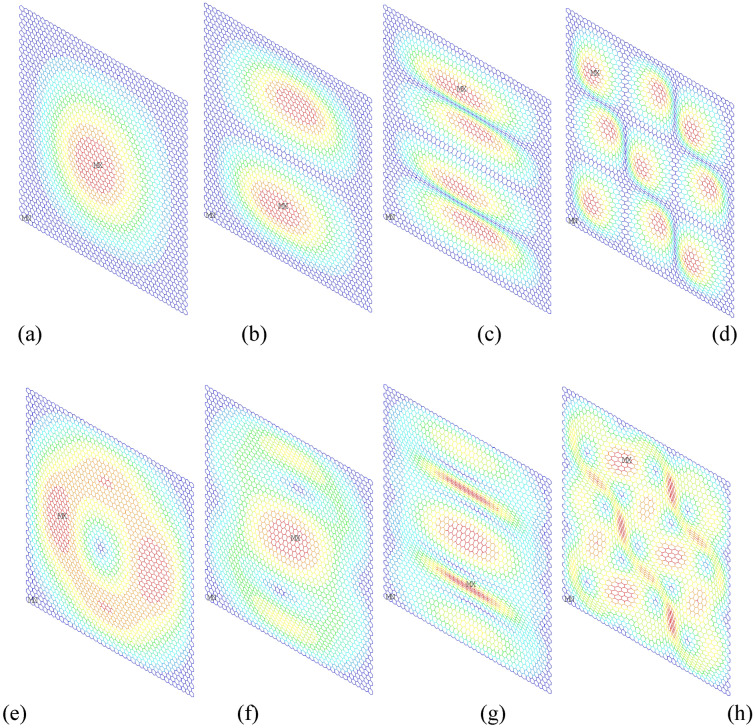
The finite element results for the deterministic FEM of graphene (**a**–**d** are the displacement vector sum of the first, second, ninth and tenth order vibrations, respectively; **e**–**h** are the rotation vector sum of the first, second, ninth and tenth order vibrations, respectively).

There are totally 6226 beams with 16,664 nodes in the deterministic FEM of graphene. As presented in Fig. 4, the displacement vector sum and roation vector sum in different vibration modes have satisfied accuracy and convergence in FEM computaion. Both the resonant frequencies and the vibration modes reach a good aggreement with the previous reported references^2,4^. Therefore, the deterministic FEM is verified as the basic model for the following MC-SFEM.

In addition, the geometrical symmetry in the displacement and rotation contour results is evident in Fig. 4. However, the resonant frequencies of graphene in different vibration orders are discrepant. The periodic honeycomb lattice of graphene with symmetrical hexagons causes the geometrical symmetry in resonant vibration modes, but the resonant frequencies are distinct to observe the resonant vibration orders. Therefore, the resonant frequencies of graphene are convenient index to track discrepances in the different resonat vibration orders, while the vibration modes such as displacement and rotation contours are senstive to the microstruture of graphene.

## Discussion and results

### Result robustness

**Figure 5:**
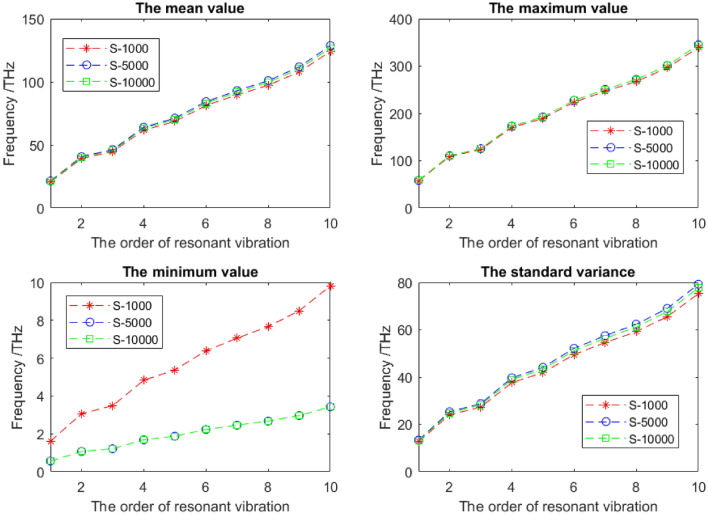
The statistic results for the resonant frequencies of graphene with different stochastic sample space sizes.

In order to record the result accuracy and convergency, the mean, maximum, minimum and standard variance values with the different stochastic sample sizes based on the MC-SFEM are recorded and compared in Fig. 5. The 10,000 groups of stochastic samples of FEM computation are supposed to be the precise results, and the compared sample space sizes are 1000 and 5000. It is evident to find that the discrepancies in the mean, maximum or standard variance values with different sample space sizes are not obvious. However, the deviations caused by the small stochastic space is observed in the minimum value of the 1000 sample groups, the statistic results of the 5000 sample groups are very approximated to that of the 10,000 sample groups. Even if the MC-SFEM has satisfied computational efficiency and accuracy, a sufficient number of stochastic samples is also a key factor. The larger sample space size requires heavier computation burden. Therefore, the trade-off between the result accuracy and computation cost exists. The discrepancies in the mean, maximum and standard variance are not as evident as that in the minimum for the size of stochastic sampling space.

**Figure 6:**
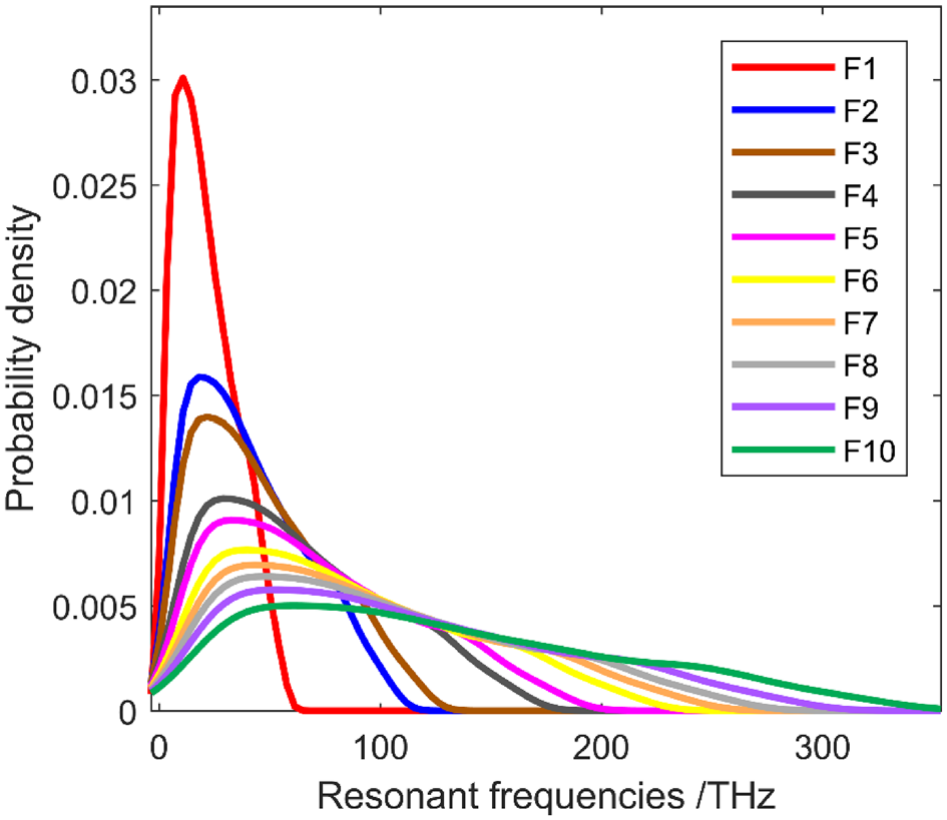
The probability density distribution for resonant frequencies of graphene (F1–F10 represent the first to the tenth order resonant vibrations).

Furthermore, the probability density distribution of the first ten resonant frequencies of graphene is also calculated according to the stochastic sampling space of MC-SFEM. The size of stochastic sampling space is 5000. Even if the characteristic parameters are distributed and sampled by the uniform distribution type, the probability density distributions of the resonant frequencies have sharp peak and long tails as shown in Fig. 6. The nonlinear relationship between the characteristic parameters and resonant frequencies are also presented in different types of probability density distributions. In addition, the right sides of the probability density distribution in Fig. 6 have wide interval ranges, which means that the resonant frequencies of graphene fluctuate within large interval ranges. Moreover, the shapes of probability density distributions of resonant frequenceis in Fig. 6 are not similar with the commonly used Gaussian, Weibull, etc., which well confirms the analytical methods by homogeneous chaos requires more further work. The explorations based on semi-analytical approaches by stochastic perturbation methods, polynomial chaos expansion, etc. are in prospect.

### Parameter comparison

**Figure 7:**
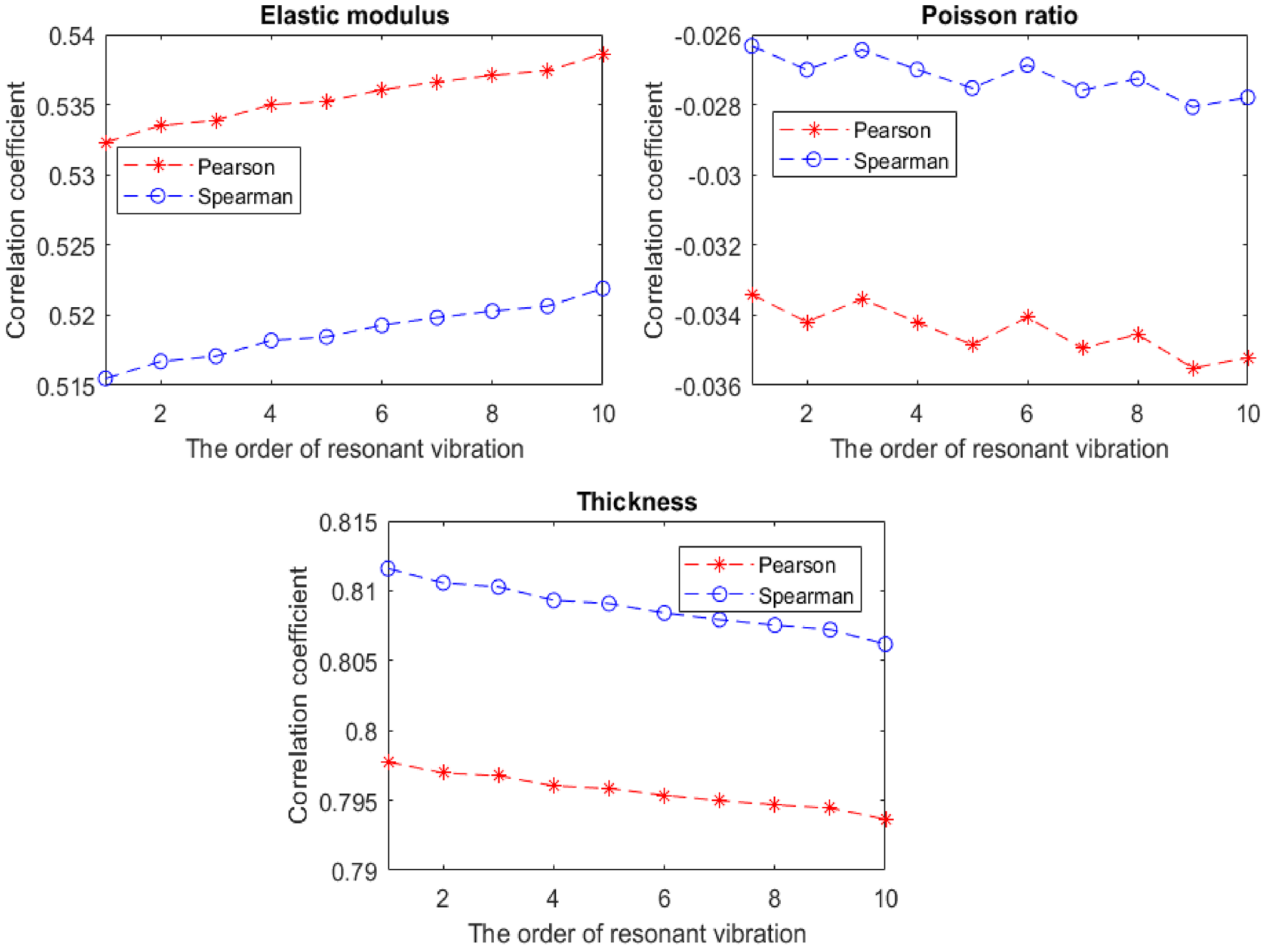
The correlation coefficient results of characteristic parameters of graphene.

Both the Pearson and Spearman correlation coefficients for the characteristic parameters and resonant frequencies of graphene are calculated and compared in Fig. 7. The Young’s modulus and thickness of graphene present positive correlation effects, while the Poisson’s ratio of graphene has negative correlation with the resonant frequencies. In addition, with the increase of resonant vibration order, the correlation coefficients of Young’s modulus increase. On the contrary, the correlation coefficients of thickness slowly decrease. However, the correlation coefficients of Poisson’s ratio not only have negative impacts but also have evident fluctuations with the increase of the resonant vibration order.

**Figure 8:**
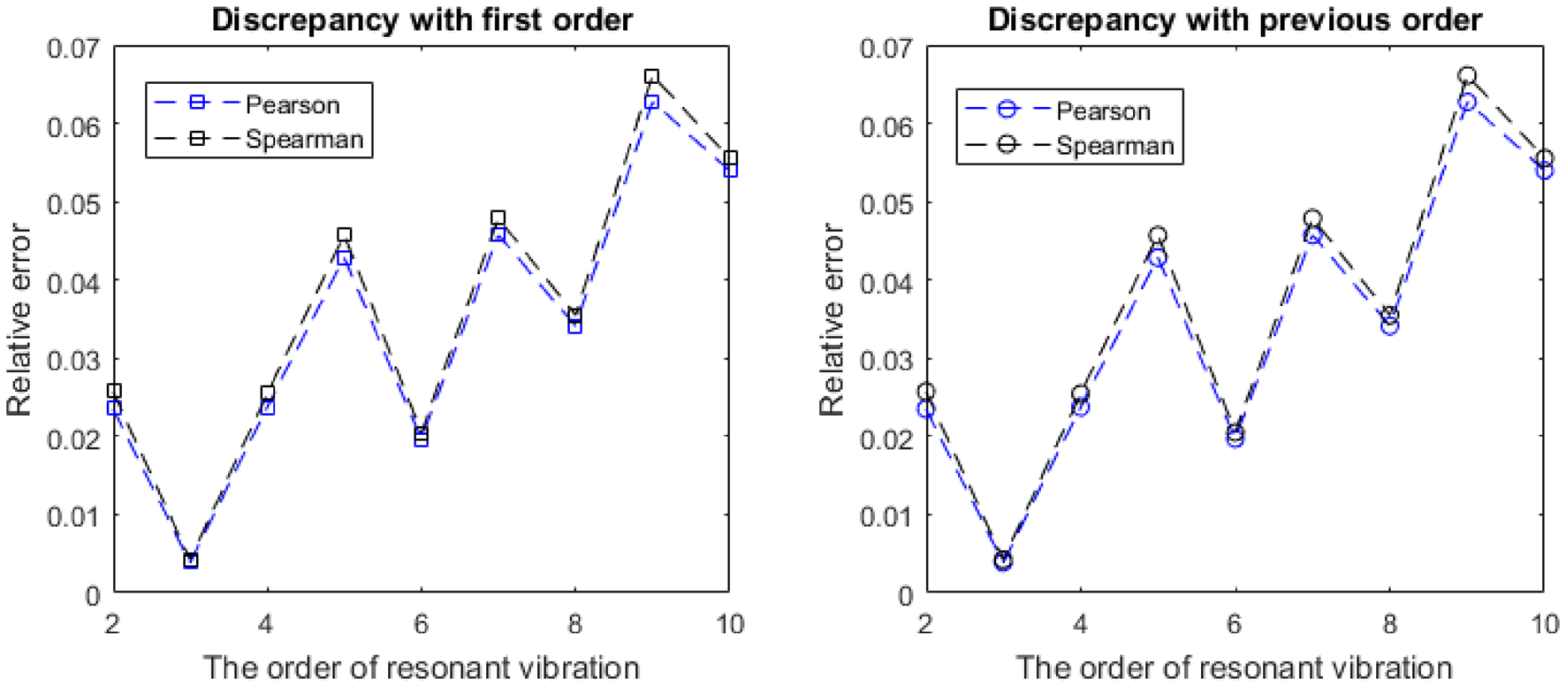
The relative errors of the Poisson’s ratio.

More precisely, the change amplitudes of the Young’s modulus are 1.18% and 1.24%, in the Pearson and Spearman correlation coefficients, respectively. From the first to the tenth vibration order, the largest reduction percentages are only 0.51% for the Pearson and 0.67% for Spearman correlation coefficients to the thickness of graphene. In terms of the Posson’s ratio, the discrepancies with the first order and previous order resonant vibrations is presented in Fig. 8. Specifically, the smallest relative error is 0.39%, while the largest relative error equals to 6.27%. In short, the correlation coefficients of Young’s modulus and thickness of graphene are settled in the narrow interval ranges with static tendancy, while the correlation coefficients of Poisson’s ratio not only have negative values but also have evident flucutations lager than 6%.

### Scatter sample results

**Figure 9:**
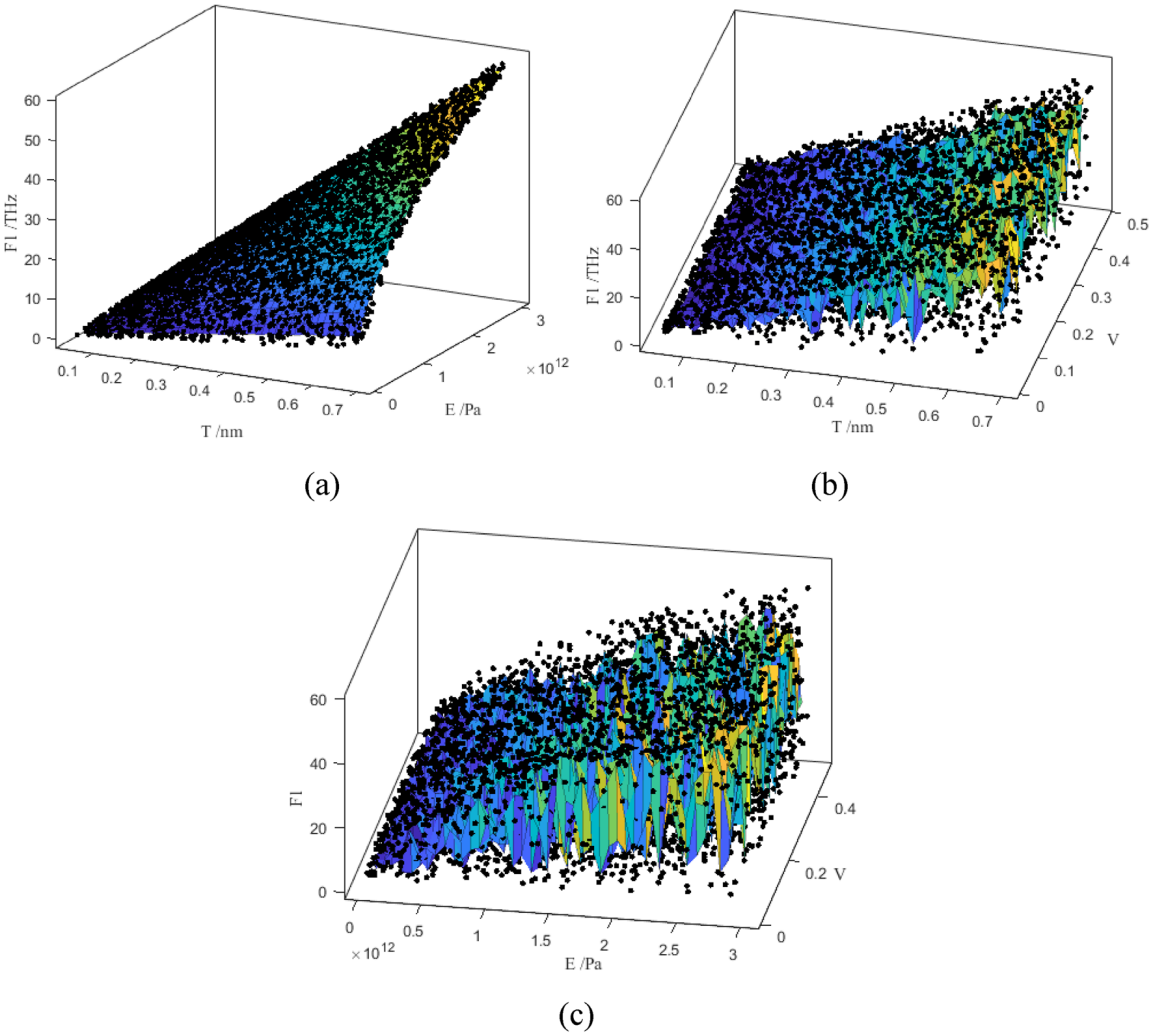
The scatter results of characteristic parameters and the first order resonant frequency of graphene (**a**–**c** represent: thickness and Young’s modulus, thickness and Poisson’s ratio, Young’s modulus and Poisson’s ratio, respectively).

In order to analyze the complicated impacts of Poisson’s ratio on the resonant frequencies, the scatter sample results are presented and compared in Fig. 9. In Fig. 9a, the thickness and Young’s modulus of graphene have the simple and linear influence on the first order resonant frequency. The results in Fig. 9b,c also prove that the Poisson’s ratio of graphene has nonlinear impacts on the first order resonant frequency. Even the correlation coefficients of Poisson’s ratio are evidently smaller than that of Young’s modulus and thickness of graphene, the nonlinear and implict relationship between the Poisson’s ratio and resonant frequencies are challenging. Therefore, the Young’s modulus and thickness of graphene are more correlated with the resonant frequencies, but the Poisson’s ratio has more implicit and nonlinear relationship with the resonant frequencies.

## Conclusion

This paper develops an efficient numerical method to propagate and quantify the uncertainty and fluctuations in the characteristic parameters of graphene. The corrlations between characteristic parameters (Young’s modulus, Poisson’s ratio, and thickness) and resonant frequencies are analyzed based on the stochastic sampling spaces created by MC-SFEM. Based on this work, the following points can be concluded.

1. The statistic results of MC-SFEM have satisfied robustness and convergence when the number of stochastic samples is large enough. However, the trade-off between result accuacy and computational cost exists.
2. The Young’s modulus and thickness of graphene have positive correlation coefficients, while the Poisson’s ratio performs the negative effects on the resonant frequencies.
3. The correlation coefficients of the Poisson’s ratio are smaller than that of the Young’s modulus and thickness of graphene, but there are evident fluctuations in both Pearson and Spearman correlation coefficients. The Poisson’s ratio is an important factor deserving more concerns.
4. The probability density distributions of resonant freqencies are far different from the commonly used probability density functions, more explorations in the semi-analytical and numerical methods are in prospect.
